# Genome-Wide Identification and Expression Profiling of the *SPL* Transcription Factor Family in Response to Abiotic Stress in Centipedegrass

**DOI:** 10.3390/plants14010062

**Published:** 2024-12-28

**Authors:** Dandan Kong, Maotao Xu, Siyu Liu, Tianqi Liu, Boyang Liu, Xiaoyun Wang, Zhixiao Dong, Xiao Ma, Junming Zhao, Xiong Lei

**Affiliations:** 1 College of Grassland Science and Technology, Sichuan Agricultural University, Chengdu 611130, China; 2 Sichuan Academy of Grassland Science, Chengdu 611731, China

**Keywords:** transcription factors, *SPL* gene family, expression profiles, *Eremochloa ophiuroides*

## Abstract

SQUAMOSA promoter-binding protein-like (SPL) transcription factors play a critical role in the regulation of gene expression and are indispensable in orchestrating plant growth and development while also improving resistance to environmental stressors. Although it has been identified across a wide array of plant species, there have been no comprehensive studies on the *SPL* gene family in centipedegrass [*Eremochloa ophiuroides* (Munro) Hack.], which is an important warm-season perennial C4 turfgrass. In this study, 19 potential *EoSPL* genes in centipedegrass were identified and assigned the names *EoSPL1*-*EoSPL19*. Gene structure and motif analysis demonstrated that there was relative consistency among the branches of the phylogenetic tree. Five pairs of segmental duplication events were detected within centipedegrass. Ten *EoSPL* genes were predicted to be targeted by miR156. Additionally, the *EoSPL* genes were found to be predominantly expressed in leaves and demonstrated diverse responses to abiotic stress (salt, drought, glufosinate ammonium, aluminum, and cold). This study offers a comprehensive insight into the *SPL* gene family in centipedegrass, creating a foundation for elucidating the functions of *EoSPL* genes and investigating their involvement in abiotic stress responses.

## 1. Introduction

The accurate modulation of gene expression is a critical mechanism that occurs throughout the lifecycle of a plant, although transcription factors (TFs) serve as pivotal regulators in the control of gene expression [1]. As key TFs in plants, SPL proteins were initially detected in the floral development of *Antirrhinum majus* [2]. SPL proteins are distinguished by the inclusion of a conserved SBP domain, which typically consists of roughly 76 amino acids (AAs) and encompasses two distinct zinc finger motifs (Cys-Cys-His-Cys and Cys-Cys-Cys-His) and a nuclear localization signal (NLS) at their C-terminus [3,4]. This domain is integral in defining the functional characteristics of SPL proteins and their involvement in diverse plant biological processes [3]. Some *SPL* gene family members are the targets of miR156, which is instrumental in regulating multiple plant developmental stages [5]. MicroRNAs (miRNAs) are brief non-coding RNA molecules, generally about 20 to 24 nucleotides in length, that function as significant regulators in post-transcriptional gene expression [5]. In particular, miR156 is implicated in a range of biological processes, such as phase transitions [6], root development [7], leaf and branch development [8,9], flower development [10], and responses to abiotic stress [11].

*SPL* genes are crucial in governing biological processes and are essential for shaping responses to abiotic stresses. In plants, *SPL* genes facilitate vegetative growth, as exemplified by *OsSPL14*-overexpressing transgenic rice (*Oryza sativa*) plants, which exhibit reduced growth duration and leaf size [12], and wheat (*Triticum aestivum*) mutants lacking *TaSPL8*, which display upright leaves [13]. *miR156/MsSPL08* regulates the number of leaflets in alfalfa (*Medicago sativa*) [14], and *OsSPL3* regulates crown root development in rice [15]. Moreover, *SPL* genes participate in reproductive growth; for instance, *TaSPL13-2B* influences floret development in wheat [16], *MsSPL20* overexpression leads to stable delayed flowering time in alfalfa [17], and *OsSPL16* controls grain size, shape, and quality [18]. *TaSPL13* mutants increase grain size and number [19], while the *Bdsbp9* mutant reduces spike length in *Brachypodium distachyon* [20]. Additionally, *SPL* genes are essential in orchestrating the transition from the vegetative to the reproductive phase in plants. For instance, the collaborative function of *SPL3/4/5* in conjunction with the FT-FD complex is pivotal for initiating flowering in *Arabidopsis* [21]. In switchgrass, the suppression of *SPL7* and *SPL8* gene expression leads to a reversal from a vegetative to a reproductive state within the inflorescence [22]. MiR156 modulates miR172 expression through *SPL* TFs, thereby promoting epidermal identity in adult plants [23]. *SPL* genes also contribute to the regulatory framework that controls plant reactions to abiotic stress conditions. For example, *AtSPL9* activates the expression of *CBF2* to increase freezing tolerance [24]. *MiR156*-overexpressing alfalfa and *SPL13* RNAi lines display enhanced heat stress tolerance, while *SPL9*-RNAi alfalfa exhibits improved drought tolerance [25,26]. Moreover, *OsSPL10* has been shown to negatively regulate salt tolerance in rice [27].

Centipedegrass [*Eremochloa ophiuroides* (Munro) Hack.] is a warm-season (C4) grass of the Poaceae family (2n = 2x = 18) and is frequently employed as a low-maintenance turfgrass [28,29]. It has a widespread distribution, including regions such as the south of the Yangtze River in China, Southeast Asia, the eastern and southern United States, and tropical northern and eastern Australia [28]. It has highly developed stolons that are extensively used for greening in courtyard and roadside areas, as well as for forage [30]. Additionally, its proficiency in tolerating and absorbing heavy metals renders it appropriate for environmental cleanup purposes [31].

The *SPL* gene family has been discovered across a diverse range of plant species, with varying numbers in different genera, including 16 *SPLs* in *Arabidopsis*, 19 in rice [32], 56 in wheat [33], 31 in maize (*Zea mays*) [34], 17 in orchardgrass (*Dactylis glomerata* L.) [35], and 76 in alfalfa [36]. Furthermore, several *SPL* genes contain conserved miR156 binding sites, as exemplified by the identification of 11 *OsSPL* genes in rice [37], 10 *SBP* (*SPL*) genes in tomato (*Solanum lycopersicon*) [38], and 12 *SBP* (*SPL*) genes in grape (*V. vinifera*) [39]. However, there are no comprehensive reports available on the *SPL* gene family in centipedegrass, which constrains our understanding of the functions and roles of *SPL* genes in centipedegrass. In this study, 19 putative members of the *SPL* gene family were discovered within the centipedegrass genome [40]. Additionally, the expression patterns of *EoSPLs* under various stress conditions (salt, polyethylene glycol, aluminum, low temperature, and glufosinate ammonium) were examined using qRT-PCR. The results not only provide valuable *SPL* family genes for molecular breeding in centipedegrass but also establish a fundamental framework for the functional investigation of *SPL* genes in various grass species.

## 2. Results

### 2.1. Identification of SPL Genes and the Chromosomal Distribution in Centipedegrass

**Figure 1:**
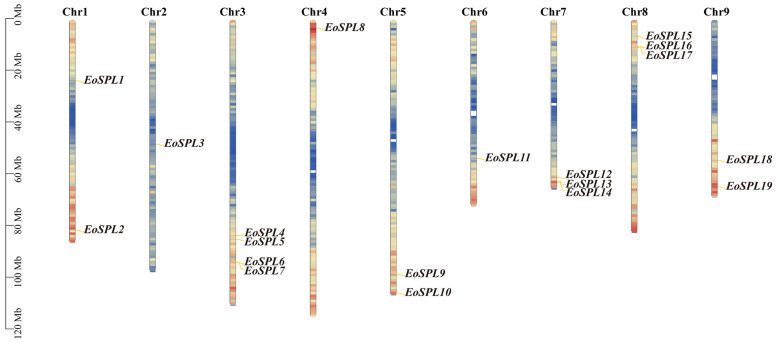
The distribution of 19 *EoSPLs* across the chromosomes. The colors on the chromosomes reflect varying levels of gene density. Blue to red colors within the chromosomes indicate increased gene density. Each chromosome is accompanied by its respective number, and the genes are labeled on the right side of the respective chromosomes for clarity.

The identification of the *SPL* genes in centipedegrass serves as the foundation for investigating the *EoSPL* gene family. A total of 19 *SPL* genes were identified in centipedegrass by excluding genes with incomplete SBP domains. These genes were designated as *EoSPL1* to *EoSPL19*, following their chromosomal locations for nomenclature consistency (Figure 1). Chr3 contained the highest number of *EoSPL* genes, including *EoSPL4*, *EoSPL5*, *EoSPL6*, and *EoSPL7*, while individual genes were located on Chr2, Chr4, and Chr6. Most *SPL* genes are positioned in regions of elevated gene density.

Molecular weights and theoretical pI values are fundamental physicochemical properties of proteins, which are particularly important in protein function research. Comprehensive information about the *EoSPL* genes is presented in Table 1. The molecular weights of the 19 EoSPL proteins ranged from 21,940.04 Da (EoSPL7) to 122,131.35 Da (EoSPL13). The theoretical pI of these SPL sequences ranged from 5.49 (EoSPL11) to 10.36 (EoSPL6). Except for EoSPL10, all SPL proteins displayed an instability index greater than 40, indicating that they may be unstable. The negative GRAVY (grand average of hydropathicity) values suggest that all proteins are hydrophilic. Subcellular localization prediction suggests that most of the *SPL* genes, excluding *EoSPL8* and *EoSPL13*, are likely to be located in the nucleus.

### 2.2. Sequence Alignments and Phylogenetic Analysis of EoSPL Proteins

**Figure 2:**
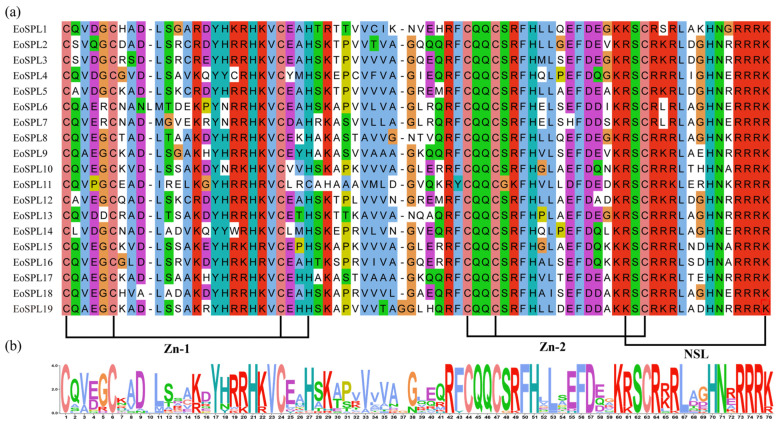
The multiple alignment (**a**) and sequence logo (**b**) of the SBP domains in the 19 EoSPL proteins.

To pinpoint the conserved domains in the *EoSPL* family, a comparative analysis of the 19 EoSPL proteins was carried out using multiple sequence alignment (Figure 2a). This analysis revealed a high degree of conservation in CQQC, SCR, and RRR sequences (Figure 2b). The SBP domains included about 76 amino acid residues, containing two zinc finger motifs (Zn-1, Zn-2) and an NLS.

**Figure 3:**
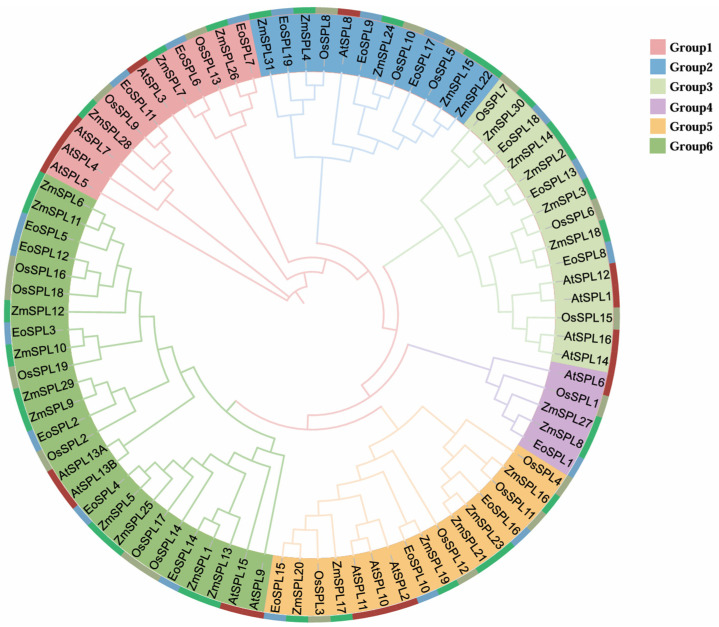
Phylogenetic tree analysis of the SPL proteins in centipedegrass, *Arabidopsis*, rice, and maize. Different colored branches denote different groups, with the SPLs being divided into six clusters based on the clustering outcomes. The proteins from centipedegrass, *Arabidopsis*, rice, and maize are depicted in blue, red, green-gray, and green, respectively.

Genes closely related to evolution may have similar functions. To explore the evolutionary relationship of the *SPL* gene family, the protein sequences of SPLs from centipedegrass, *Arabidopsis*, rice, and maize were used to construct the phylogenetic tree (Figure 3). The EoSPL proteins were classified into six unique groups (Group 1 to Group 6), with each group harboring at least one EoSPL protein. The most extensive group (Group 6) comprised 26 proteins. In the phylogenetic tree, all EoSPL proteins exhibit closer evolutionary relationships with members of maize, indicating that the functions of some *SPL* genes in centipedegrass can be inferred from homologous genes in maize.

### 2.3. Gene Structure and Conserved Motif Analysis of EoSPL Genes

**Figure 4:**
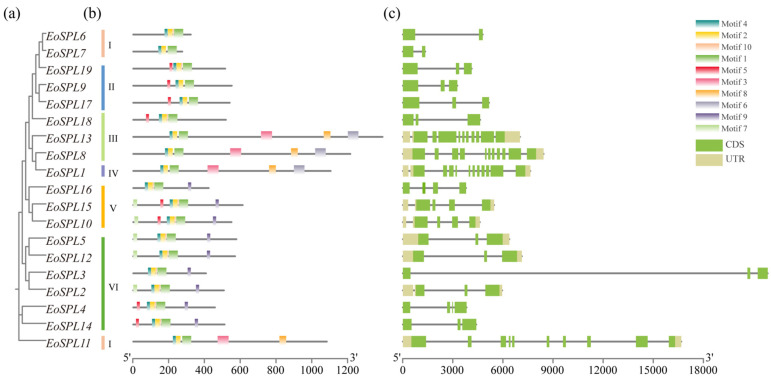
Phylogenetic relationships, motifs, and gene structures in *EoSPL* genes. (**a**) The phylogenetic tree was constructed based on the EoSPL protein sequences using TBtools-II. (**b**) The motif composition of the EoSPL proteins was performed using TBtools-II. (**c**) Gene structures of the *EoSPL* genes. Green boxes indicate CDS regions; grey boxes indicate UTR regions. I-VI is the grouping of EoSPL proteins in the phylogenetic tree.

An analysis of gene structure and motif variety elucidated the evolutionary dynamics of the gene family. Among the 19 *EoSPL* genes in the phylogenetic tree, Group 6 had the most members, comprising six genes (Figure 4a). All *EoSPL* genes contained more than three conserved motifs, and motif 6 was exclusively found in *EoSPL1*, *EoSPL8*, and *EoSPL13* (Figure 4b). *EoSPL1* possesses 11 introns, whereas *EoSPL6* and *EoSPL7* each have 1 intron (Figure 4c). Moreover, nine *EoSPL* genes displayed the presence of both 5′-UTR and 3′-UTR regions (Figure 4c). It is evident that *EoSPLs* grouped on the same branch share comparable structures and conserved motifs. The differences in the structure of *EoSPL* genes are the foundations for the diversity in gene functions, indicating the intricate nature of the function of SPL proteins in centipedegrass.

### 2.4. Duplication Analysis of EoSPL Genes

**Figure 5:**
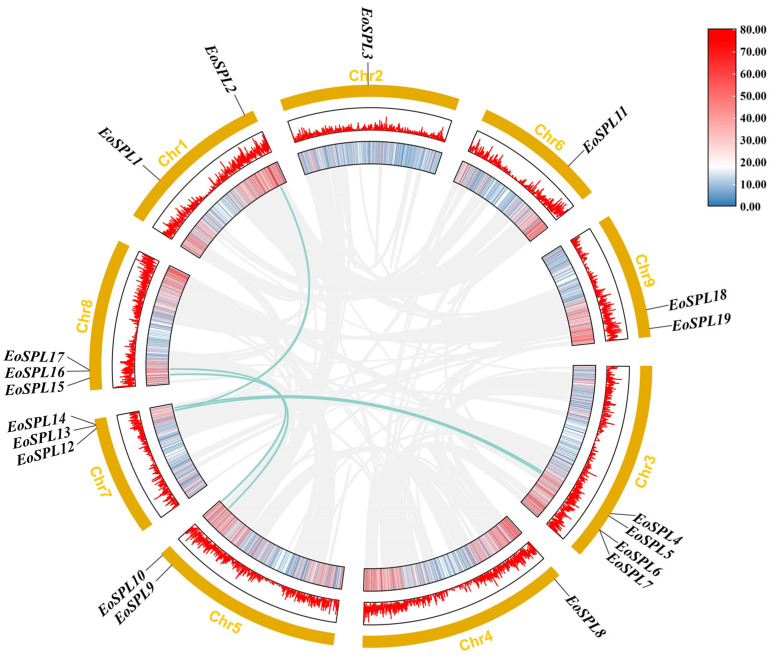
Gene duplications of the *EoSPLs*. The gray lines represent all synthetic blocks in the genome, whereas the chromatic line denotes the segmental duplication events in the *EoSPL* gene family.

In centipedegrass, six pairs of genes were identified as gene duplications within the *EoSPL* gene family (Figure 5 and Table 2), including one pair of tandem duplication events (*EoSPL6*/*EoSPL7*) and five pairs of segmental duplication events (*EoSPL2*/*EoSPL12*, *EoSPL4*/*EoSPL14*, *EoSPL5*/*EoSPL12*, *EoSPL9*/*EoSPL17*, and *EoSPL10*/*EoSPL15*). To better understand the selective pressures on these duplicated genes, we determined the values of Ka, Ks, and the corresponding Ka/Ks ratios (Table 2). The analysis revealed that the *EoSPL* family exhibited low Ka/Ks values (<1) for all duplicated pairs, indicating that these genes may be present under strong purifying selection. Additionally, the gene duplication times ranged from 23.3462 to 82.2308 MYA (million years ago) (Table 2).

### 2.5. Collinearity Analysis of EoSPL Genes Between Centipedegrass and Other Species

**Figure 6:**
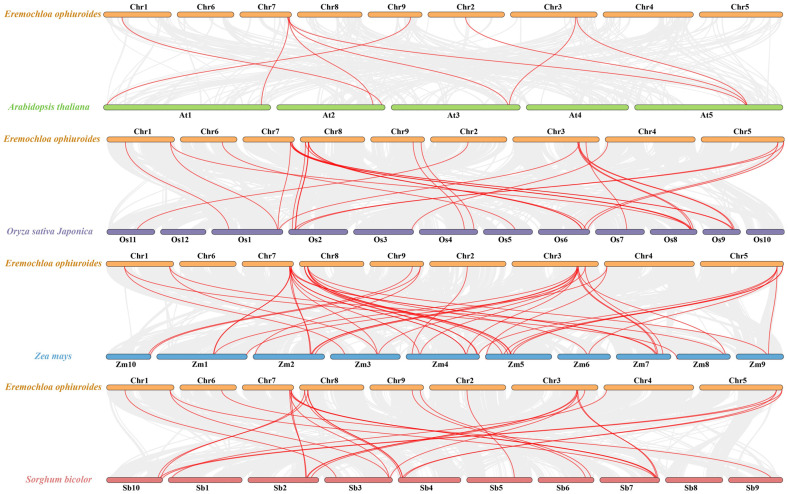
Synteny analysis between the *SPL* genes of centipedegrass and four model plant species. The gray lines in the background signify collinear blocks within the genomes of centipedegrass and the other plants, while the red lines emphasize the syntenic *SPL* gene pairs.

To investigate the syntenic connections of *EoSPL* genes across different species, a comparative synteny analysis was performed using four exemplary plants. This involved *Arabidopsis* and three members of the Poaceae family: rice, maize, and sorghum (*Sorghum bicolor*). The total of *SPL* orthologous gene pairs for centipedegrass with each of these species was 9 for *Arabidopsis*, 30 for rice, 45 for maize, and 29 for sorghum (Figure 6). The results showed that the phylogenetic relationships between centipedegrass and rice, maize, and sorghum were closer than those with *Arabidopsis*.

### 2.6. Analysis of Cis-Acting Elements in the Promoter of EoSPL Genes

**Figure 7:**
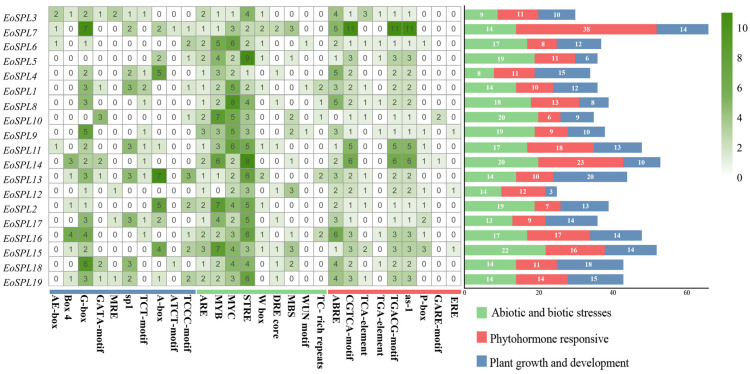
*Cis*-elements of the *EoSPLs*. The color gradient from lighter to dark green represents the number of elements from lower to higher.

To understand the likely roles of *EoSPLs*, we performed an investigation of the *cis*-acting elements within the promoters of 19 *EoSPL* genes (Figure 7). Elements related to responses to abiotic and biotic stresses were most frequently found, indicating that *EoSPLs* could be crucial in mediating stress responses. The findings showed that nine *EoSPL* genes contained MBS (a drought response element), five contained TC-rich repeats (defense and stress response elements), and three contained WUN motifs (wound response elements). Interestingly, all *EoSPL* genes contained anaerobic induction-responsive elements. In addition, most of the *cis*-elements associated with plant hormone reactivity were found in *EoSPL7*, among which MeJA response elements were the most abundant.

### 2.7. Prediction of the miR156 Target Sites in the EoSPL Gene

**Figure 8:**
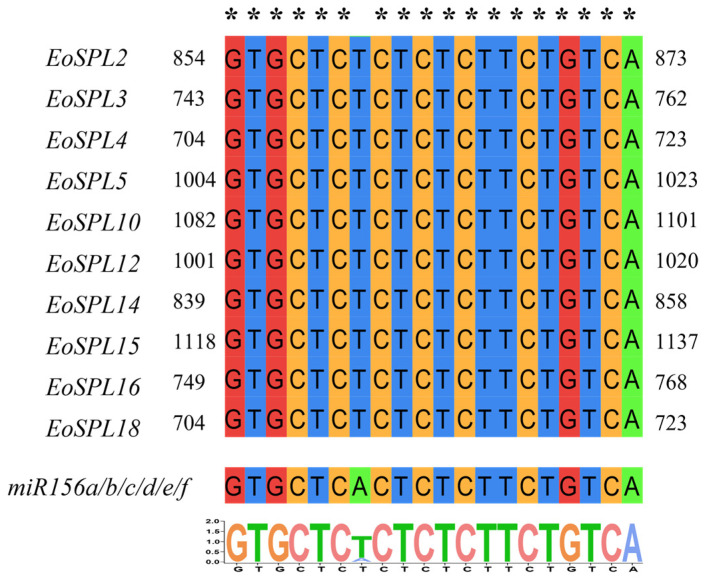
Multiple alignments of *EoSPL* genes and *miR156* complementary sequences. The target locations were determined through the coding regions of the *EoSPL* genes using the psRNATarget (https://www.zhaolab.org/psRNATarget/, accessed on 2 October 2024), while the mature sequence of *miR156* was obtained from the miRBase database (https://www.mirbase.org/, accessed on 2 October 2024).

To enhance our understanding of the miR156-mediated regulation of the expression of *EoSPLs* at the post-transcriptional level, a multiple sequence alignment analysis was conducted by identifying potential target sites of miR156 within the coding regions of the *EoSPLs* (Figure 8). The results show that 10 *EoSPL* genes had target sites in the coding region, indicating that miR156 may have a potential regulatory effect on these *EoSPL* genes. This study found that miR156 influenced the coding regions of *EoSPL2*, *EoSPL3*, *EoSPL4*, *EoSPL5*, *EoSPL12*, and *EoSPL14*, which belong to Group 6. Additionally, it influenced *EoSPL10*, *EoSPL15*, and *EoSPL16* from Group 5 and *EoSPL18* from Group 3. The findings imply that the post-transcriptional regulation of *SPLs* by miR156 is a conserved characteristic among plant species.

### 2.8. Analysis of the Protein–Protein Interaction Network of EoSPL Proteins

**Figure 9:**
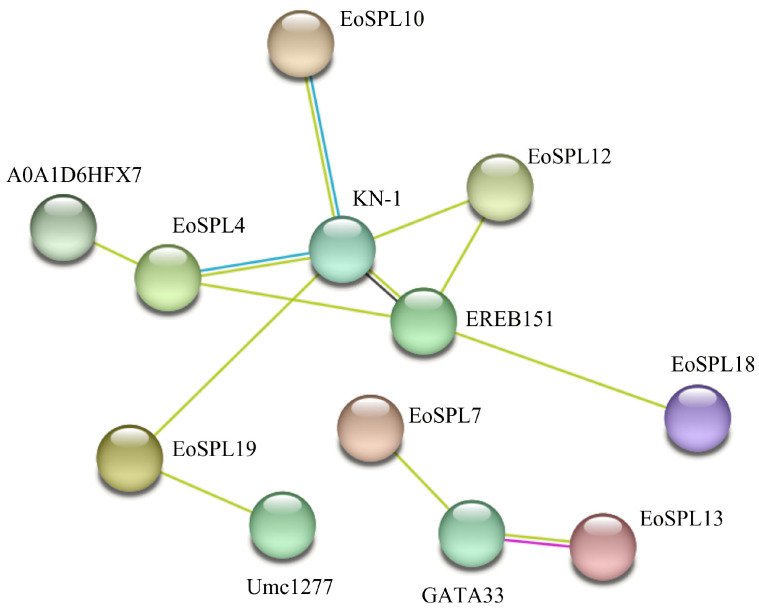
The PPI network of EoSPL proteins and other proteins. Different colors and positions represent the degree of interaction with EoSPL proteins, with proteins closer to the EoSPL circle showing stronger interactions.

To delve deeper into the potential interactions between EoSPL proteins and other proteins, we performed PPI predictions. A total of five proteins (A0A1D6HFX7, EREB151, Umc1277, GATA33, and KN-1) were identified as having strong interactions with EoSPL proteins (Figure 9). EREB151 belongs to the AP2-EREBP transcription factor family, which suggests that there is likely to be an interaction between the EoSPL proteins and the AP2-EREBP protein. The robust interaction between SPL proteins and other proteins suggests that they may share common functional roles.

### 2.9. Expression Analysis of EoSPL Genes in Different Tissues

**Figure 10:**
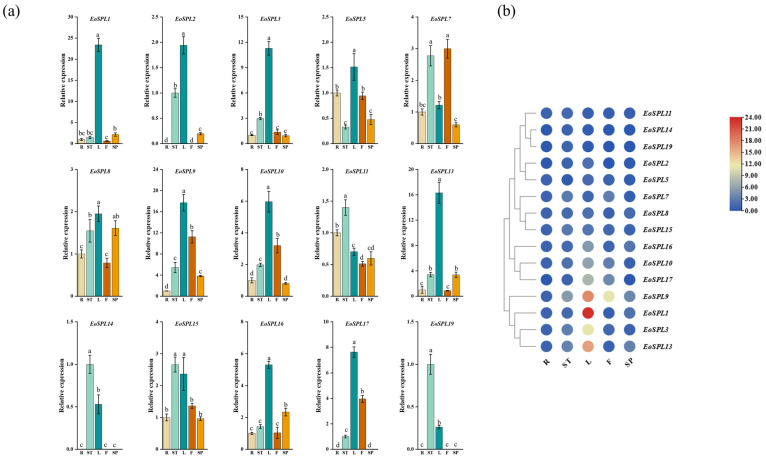
The expression levels of *EoSPLs* across various plant tissues. (**a**) The expression levels of 15 *EoSPL* genes were examined using a qRT-PCR assay. Error bars indicate the standard deviation (SD) derived from three replicates. Lowercase letter(s) above the bars indicate significant differences (α = 0.05, LSD) among various plant tissues. (**b**) Expression values are based on qRT-PCR and visualized using TBtools-II. High expression levels are represented by red, while low expression levels are indicated by blue. R: root; ST: stem; L: leaf; F: flower; SP: spike.

The data from the qRT-PCR experiments were analyzed to provide insights into the tissue-specific expression patterns of 15 *EoSPL* genes (Figure 10, Table S1). The results revealed that *EoSPL1*, *EoSPL2*, *EoSPL3*, *EoSPL5*, *EoSPL8*, *EoSPL9*, *EoSPL10*, *EoSPL13*, *EoSPL16*, and *EoSPL17* displayed a preference for expression in leaves. Conversely, the relative expression levels of *EoSPL11*, *EoSPL14*, *EoSPL15*, and *EoSPL19* were higher in stems, and *EoSPL7* was most abundant in flowers. These findings suggest a broad role for *EoSPL* genes in the growth and developmental processes of centipedegrass.

### 2.10. Expression Analysis of EoSPL Genes Under Different Abiotic Stresses

**Figure 11:**
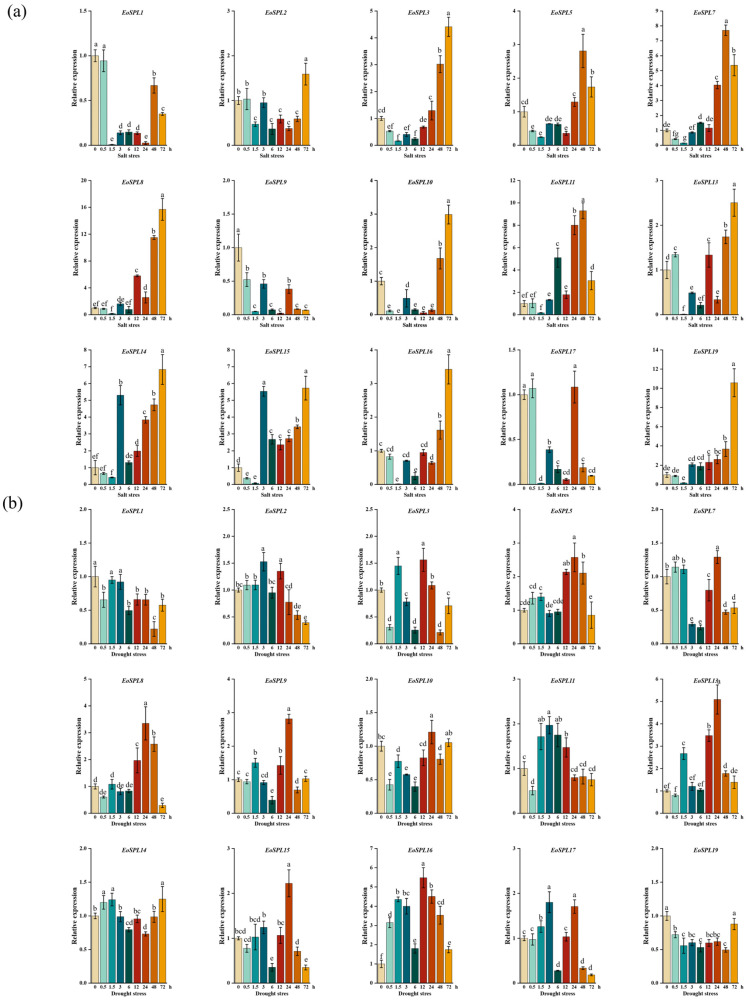

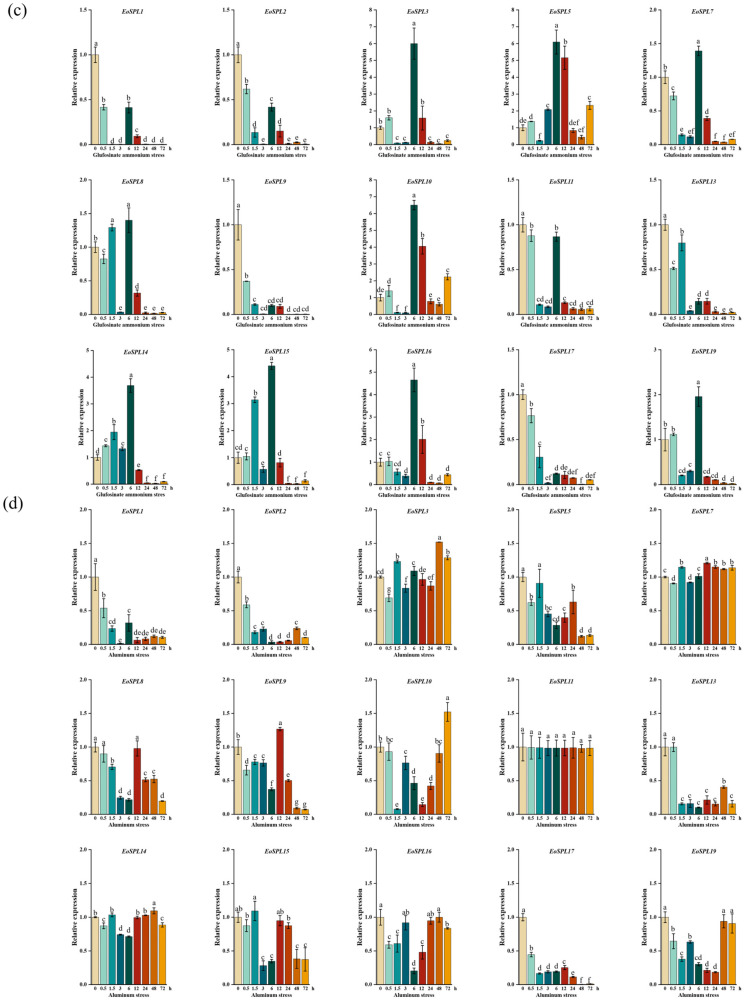

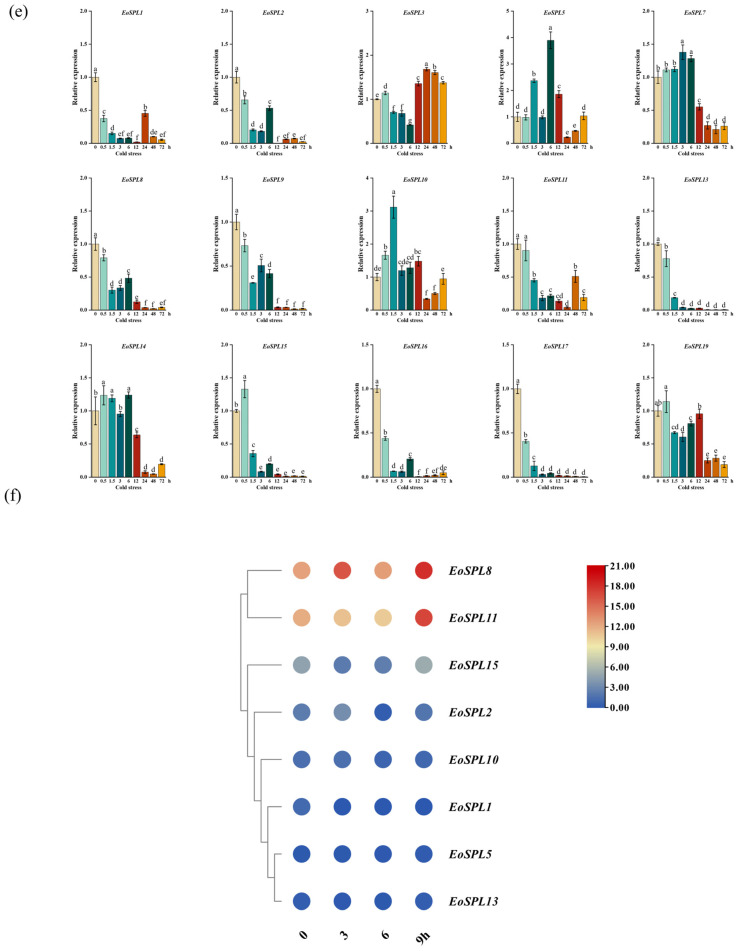
Relative expression of *EoSPLs* under abiotic stresses: (**a**) salt stress; (**b**) drought stress; (**c**) glufosinate ammonium stress; (**d**) aluminum stress; (**e**) cold stress using a qRT-PCR assay; and (**f**) cold stress expression profiles of 8 *EoSPL* genes from RNA-seq. The expression levels of 15 *EoSPL* genes in leaves after 0 h (CK), 0.5 h, 1.5 h, 3 h, 6 h, 9 h, 12 h, 24 h, 48 h, and 72 h of different abiotic stress treatments was examined through qRT-PCR. Error bars indicate the standard deviation (SD) derived from three replicates. Lowercase letter(s) above the bars indicate significant differences (α = 0.05, LSD) among stress treatments.

To study how *EoSPL* genes are expressed under various abiotic stress situations, the expression levels of 15 *EoSPLs* across five experimental conditions were quantified using qRT-PCR (Figure 11). Under the salt treatment, the expression patterns of the majority of the *EoSPL* genes displayed an upward trend as processing time increased (Figure 11a). Among them, *EoSPL2*, *EoSPL3*, *EoSPL8*, *EoSPL10*, *EoSPL13*, *EoSPL14*, *EoSPL16*, and *EoSPL19* exhibited higher expression levels at 72 h under salt stress conditions. Conversely, the relative expression levels of *EoSPL5*, *EoSPL7*, and *EoSPL11* were higher at 48 h but decreased with the increase in salt stress time. The results indicated that *EoSPL* genes responded positively to salt stress. Moreover, the expression levels of *EoSPL1* and *EoSPL9* were reduced at each time point compared to the control conditions under salt stress.

Focusing on the drought treatment, a difference in expression profiles was observed when comparing treatment conditions to the control group (Figure 11b). It can be seen that the expression patterns of *EoSPL2*, *EoSPL11*, and *EoSPL17* were increased at 3 h, and *EoSPL5*, *EoSPL7*, *EoSPL8*, *EoSPL9*, *EoSPL10*, *EoSPL13*, and *EoSPL15* were upregulated at 24 h under drought stress. Further analysis revealed that *EoSPL3* and *EoSPL16* exhibited augmented expression levels at 12 h, with these levels initially rising before subsequently declining as the drought stress time increased. Intriguingly, the expression levels of *EoSPL1* and *EoSPL19* were lower than those of the control under drought stress conditions.

Under glufosinate ammonium stress, the expression levels for *EoSPL1*, *EoSPL2*, *EoSPL9*, *EoSPL11*, *EoSPL13*, and *EoSPL17* were reduced when compared to the control, and the expression levels for the other genes were the highest at 6 h of treatment (Figure 11c). Additionally, the expression levels for *EoSPL1*, *EoSPL2*, *EoSPL5*, *EoSPL8*, *EoSPL13*, *EoSPL17*, and *EoSPL19* were lower in comparison to the control under aluminum stress. The expression levels for *EoSPL3*, *EoSPL9*, and *EoSPL10* were higher at 48 h, 12 h, and 72 h under aluminum stress, respectively, whereas the other genes exhibited minimal changes (Figure 11d).

The expression levels of *EoSPL3*, *EoSPL5*, *EoSPL7*, and *EoSPL10* were determined following cold stress, with *EoSPL3* reaching its peak expression at 24 h, *EoSPL5* at 6 h, *EoSPL7* at 3 h, and *EoSPL10* at 1.5 h (Figure 11e). Moreover, the expression of *EoSPL14* and *EoSPL19* decreased with the increase in cold stress time. To further investigate the functions of *EoSPL* genes in coping with cold stress, the expression profiles of selected genes were examined utilizing RNA-seq data obtained from experiments conducted at low temperatures. The expression patterns of eight *EoSPLs* under cold stress were visualized in a heatmap (Figure 11f). The expression patterns of *EoSPL1* were consistently lower than the control across all time points. The expression levels of *EoSPL2* and *EoSPL10* reached their peak at 3 h, after which they progressively decreased. The expression patterns of *EoSPL5*, *EoSPL11*, *EoSPL13*, and *EoSPL15* exhibited a trend of initial decrease followed by an increase. The expression pattern of *EoSPL8* demonstrated an initial rise, a subsequent decrease, and a final increase. The results were generally consistent with our qRT-PCR results and confirmed the reliability of the qRT-PCR results.

## 3. Discussion

The *SPL* family represents a distinctive group of transcription factors unique to plants, which have been identified and extensively studied in organisms such as *Arabidopsis*, rice [32], maize [34], and numerous additional plant species [36,38,39]. The highly conserved SBP domain is a defining feature of the *SPL* family [3,4], which is consistent with our research findings (Figure 2). A total of 10 *EoSPL* genes in centipedegrass possessed target sites of miR156, suggesting that the *EoSPL* genes may play a role in the *mi156/SPL* module (Figure 8). Interestingly, most *miR156*-targeted *SPL* genes were clustered within a class, such as *EoSPL10*, *EoSPL15*, *EoSPL16*, *OsSPL12*, *AtSPL2*, *AtSPL10*, and *AtSPL11* in Group 5 (Figure 3). The findings indicated that *SPL* genes targeted by *miR156* are strongly conserved across plant species, which contributes to our understanding of the evolutionary processes of these genes.

The variation in the count of *SPL* genes among different plant species indicates that the evolution of these genes has been significantly shaped by various gene duplication events. Gene duplications are recognized as significant mechanisms that drive the diversification and expansion of gene families [41]. Segmental duplications are a significant source of genetic variation, considered as one of the main drivers of the expansion of plant gene families [42]. The amplification of gene families during plant evolution is primarily attributable to frequent tandem duplication events [41]. In our study, segmental duplication was the primary mechanism behind the evolutionary growth of the *EoSPL* family. These segmental duplication events contributed to the formation and expansion of the *EoSPL* gene family. Among these pairs, six had Ka/Ks values of <1, indicating that purifying selection has played a significant role in shaping the evolution of these genes. These results align with previous studies on *SPL* genes in wheat [33], suggesting that the evolution of *EoSPLs* is comparable to that of *SPL* genes in other plant species. Additionally, the number of orthologous gene pairs was determined between centipedegrass and four other plant species (*Arabidopsis*, rice, maize, and sorghum) to deduce the functions of specific *EoSPLs*. The number of orthologous gene pairs indicated that there is a strong relationship between *EoSPLs* and *ZmSPLs*, *OsSPLs*, and *SbSPLs* compared to *AtSPLs*. Therefore, it can be concluded that the evolutionary trajectory of the *SPL* gene family in centipedegrass exhibits similarities to that in other plants of the Poaceae family. The phylogenetic analysis revealed that the *SPL* genes of different plant species were functionally conserved. *OsSPL14* exhibits a close evolutionary relationship with *EoSPL4* and *EoSPL14* (Figure 3). This indicates that these *EoSPL* genes may be potentially associated with leaf development, as miR156-targeted *OsSPL14* was involved in reduced growth duration and leaf size [12]. Moreover, the loss of function of *OsSPL14/17* in the rice mutant of *OsMADS5* abrogated its promotional effect on seminal root elongation under NH_4_^+^ conditions [43], suggesting an alternative avenue for investigating the role of *EoSPL4* and *EoSPL14*. Our results showed that all *EoSPL* genes contain 1–11 introns, and the introns exhibit wide variability (Figure 4). This characteristic is reminiscent of the high intron variability observed in *SPL* gene family members in maize [34]. Additionally, motifs 1, 2, and 4 were identified within the 19 EoSPL proteins, indicating that these motifs are essential for their function as TFs (Figure 4). Furthermore, our results revealed that evolutionarily related *EoSPLs* possessed similar motifs and exon/introns. These results not only support the accuracy of the constructed evolutionary tree of EoSPL proteins but also offer further proof of the conservation of the *SPL* gene throughout evolutionary history.

Promoters, along with their associated *cis*-acting elements, play a crucial role in the transcriptional regulation of genes [44]. Our research revealed various *cis*-elements that are known to participate in biotic and abiotic stress responses, growth and development, and phytohormone regulation (Figure 7). Notably, the abiotic and biotic stress elements were found to be the most prevalent, suggesting that *EoSPL* genes play a significant role in plant responses to stress. Specifically, two elements (CGTCA/TGACG motifs) were linked to the response to MeJA, whereas another two elements (P-box and GARE motifs) were associated with gibberellin responsiveness. Furthermore, all genes but *EoSPL10* included ABRE elements, which are pertinent to ABA responsiveness. ABRE-binding proteins (AREBs) or ABRE-binding factors (ABFs) can bind to the ABRE elements of ABA-responsive genes to induce their expression [45]. This indicated that the majority of *EoSPL* genes could be engaged in abiotic stress responses since ABA is a renowned stress-related plant hormone [45]. Additionally, CBFs (C-repeat binding factors) bind to the DRE (dehydration-responsive element) of the *COR* (cold-responsive) genes to activate their expressions for increasing cold tolerance in plants [24,46]. In this study, nine *EoSPL* genes (*EoSPL4*, *EoSPL7*, *EoSPL8*, *EoSPL11*, *EoSPL12*, *EoSPL17*, *EoSPL15*, *EoSPL18*, and *EoSPL19*) included DRE elements, which may be associated with cold stress.

The expression patterns of *EoSPL* genes in various tissues provide insights into their potential involvement in biological processes. In our study, the expression levels of the 15 *EoSPLs* exhibited tissue-specific variation (Figure 10). This variation suggests that *EoSPL* genes may be involved in various aspects of centipedegrass development. The expression of *EoSPL* genes was predominantly high in leaves (Figure 10), indicating that the *EoSPL* gene may play a primary role in leaf development. miR156-targeted *EoSPL2*, *EoSPL3*, *EoSPL5*, *EoSPL10*, and *EoSPL16* exhibited elevated expression levels in leaf tissue, suggesting that these genes have a potential role in centipedegrass leaf development. The expression of four genes (*EoSPL11*, *EoSPL14*, *EoSPL15*, and *EoSPL19*) in stems was higher, while the expression of one gene (*EoSPL7*) in flowers was higher (Figure 10), indicating that *EoSPL* genes had distinct roles in stem and flower development. In *Arabidopsis*, *AtSPL15* and *AtSPL9* loss-of-function results in a shortened plastochron during vegetative growth [47]. *EoSPL14*, which shares a clade with these genes, was found to be expressed at a lower level in leaves compared to stems in our study (Figure 3). This discovery aligns with published reports that the *SPL* gene plays a role in the regulation of flower [17] and leaf development [12]. In conclusion, the expression levels of *EoSPLs* across different tissues in centipedegrass showed that they were involved in the complex mechanisms underlying growth and development. Moreover, these findings corroborate previous studies on the functions of *SPLs* and suggest that the *EoSPL* family may have evolved similar functions in centipedegrass. Therefore, it is imperative to delve deeper into the specific molecular mechanisms through which the *EoSPL* gene orchestrates these developmental processes in future research endeavors.

It is a significant aspect of their function in plant biology that *SPL* genes confer tolerance to various abiotic stresses [25,26,27]. The expression levels of 15 *EoSPL* genes were up- or downregulated in response to five different stresses, suggesting that they were involved in abiotic stress (Figure 11). In rice, *OsSPL10* has been shown to negatively regulate salt tolerance [27]. Furthermore, there is a close evolutionary relationship between *EoSPL9*, *EoSPL17*, *EoSPL19*, and *OsSPL10*. The expression of *EoSPL9* in centipedegrass was lower than that of the control at each time point under salt stress (Figure 11a). Furthermore, knockdown and knockout of *OsSPL10* in rice were found to enhance drought tolerance [48], and our results showed that the expression of *EoSPL19* was also lower than that of the control at each time point under drought stress (Figure 11b). Thus, it is hypothesized that *EoSPL9* and *EoSPL19* may have similar functions in centipedegrass. Additionally, *AtSPL9* has been reported to activate *CBF2* expression and increase freezing tolerance in *Arabidopsis* [24]; *EoSPL14* has a close evolutionary relationship with *AtSPL9*. In addition, the expression of *EoSPL14* was higher than that of the control at multiple time points under cold stress (Figure 11e). These results suggested that the *EoSPL* gene family plays a complex role in plant stress tolerance, with individual genes potentially serving as either positive or negative regulators depending on the specific stress. Hence, it is of great significance to understand the molecular mechanisms of the differential regulation of *EoSPL* genes in response to abiotic stress in order to formulate strategies to improve the stress resistance of turfgrass species.

## 4. Materials and Methods

### 4.1. Identification of the SPL Gene Family in Centipedegrass

The genomic resources for centipedegrass were obtained from the Figshare database (https://figshare.com/s/8256acffdb73bb050045, accessed on 2 October 2024) [40]. The *EoSPL* gene family members were identified in the genome of centipedegrass using the HMM (hidden Markov model) profile of the SBP domain (PF03110) retrieved from the Pfam database (http://pfam.xfam.org, accessed on 2 October 2024). We then validated these candidates by employing the Batch CD-Search Tool (https://www.ncbi.nlm.nih.gov/Structure/bwrpsb/bwrpsb.cgi, accessed on 2 October 2024) and Pfam, focusing on the integrity of the SBP domain to confirm the identity of the genes. Then, sequences lacking the typical SBP binding domain characteristic of SPL proteins were excluded. Furthermore, several protein properties of the *EoSPLs* were calculated using the ExPASy website (https://web.expasy.org/protparam/, accessed on 2 October 2024). Subcellular localization predictions for EoSPL proteins were also performed with WoLF PSORT (https://wolfpsort.hgc.jp/, accessed on 2 October 2024) [49]. Lastly, TBtools-II was utilized to map these *EoSPL* genes onto their respective chromosomes [50].

### 4.2. Sequence Alignments and Phylogenetic Analysis of EoSPL Genes

Sequence alignments of the SBP domains within the EoSPL proteins were carried out using Mafft with default settings within Jalview v2.11.2.0, and the alignments were visualized using the same software [51]. Subsequently, a sequence logo for the SBP domains was created with TBtools-II [50]. For the phylogenetic analysis, protein sequences of *Arabidopsis*, rice, and maize were obtained from PlantTFDB (http://planttfdb.gao-lab.org/, accessed on 2 October 2024) [52]. The phylogenetic trees were constructed using the neighbor-joining (NJ) approach within MEGA [53].

### 4.3. Gene Structure and Conserved Motif Analysis of EoSPL Genes

TBtools-II was employed to detect conserved motifs within the *EoSPL* genes, with the motif count capped at a maximum of 10 [50]. Furthermore, this tool was used to analyze and visualize the phylogenetic tree, conserved motifs, and gene structures of the 19 EoSPL proteins [50].

### 4.4. Gene Duplication Events and Collinearity Analysis

MCScanX was used to identify individual *EoSPL* gene duplication events throughout evolutionary history [54]. The gene duplication events of *EoSPL* genes were visually represented using TBtools-II [50]. Collinearity analysis of *EoSPL* genes between centipedegrass and other species (*Arabidopsis*, rice, maize, and sorghum) was performed using TBtools-II [50]. Furthermore, the gene duplication time was calculated according to the formula: T = Ks/2λ (λ = 6.5 × 10^−9^) [55].

### 4.5. Promoter Analysis and miR156 Target Prediction of EoSPL Genes

The 2000 bp upstream sequences of the *EoSPL* genes were submitted to PlantCARE (https://bioinformatics.psb.ugent.be/webtools/plantcare/html/, accessed on 2 October 2024) to analyze their *cis*-regulatory elements [56]. The sequences of miR156 were obtained from the miRBase database (https://www.mirbase.org/, accessed on 2 October 2024) [57]. The target locations were determined through the coding regions of the *EoSPL* genes using psRNATarget (https://www.zhaolab.org/psRNATarget/, accessed on 2 October 2024) [58].

### 4.6. Plant Material and Treatment

Seeds of the centipedegrass cultivar “Wuling” were sown in small square pots containing quartz sand within a plant growth chamber. The growth conditions for the plant material were as follows: 23 °C/19 °C (12 h day/12 h night). Seedlings were subjected to each stress after a 90-day growth period. Salt stress was induced with 200 mmol·L^−1^ NaCl irrigation, while drought stress was mimicked using 20% PEG-6000. Glufosinate ammonium was sprayed at a concentration of 6 mmol·L^−1^. Aluminum stress was applied using 100 µmol·L^−1^ AlCl_3_ solution, and cold stress was performed using an incubator set at 4 °C. Samples were harvested at 0, 0.5, 1.5, 3, 6, 12, 24, 48, and 72 h after each application of stress. Each sampling point consisted of three independent biological replicates. Then, the samples were quickly immersed in liquid nitrogen to preserve their condition and stored at −80 °C.

### 4.7. qRT-PCR Analysis of Centipedegrass

Total RNA extraction was performed using the M5 HiPer Plant Complex Mini Kit (Juhemei, Beijing, China). The extracted RNA was then transformed into cDNA using the ABScript III RT Master Mix for qPCR with gDNA Remover (Abclonal, Wuhan, China). qRT-PCR was performed using the Genious 2× SYBR Green Fast qPCR Mix (Abclonal, Wuhan, China) according to the manufacturer’s protocol and the CFX96 Realtime PCR system (Bio-Rad, Hercules, CA, USA). The experiment was carried out in a 10 µL system. The experimental protocol was as follows: an initial denaturation phase at 95 °C for a duration of 30 s. Subsequently, 40 cycles of denaturation were performed at 95 °C for 10 s by annealing at 58 °C for a duration of 10 s. Finally, a final extension step was carried out. The chosen internal reference genes were *UBC* (*ubiquitin-conjugating enzyme*) for tissues, cold, and aluminum stress, *MD* (*malate dehydrogenase*) for drought and salt stress, and *RIP* (*60S ribosomal protein L2*) for glufosinate and ammonium stress [59]. The relative expression levels of *EoSPLs* were obtained using the 2^−ΔΔCt^ method [60]. Each sampling point consisted of three independent biological replicates. A total of fifteen primer pairs specific to the *EoSPLs* were crafted using Primer 5 software, and the details of these primers are provided in Table S2.

### 4.8. Expression Profiles of EoSPLs in Cold Stress and Prediction of Protein–Protein Interactions of EoSPL Protein

Previous RNA-seq data were employed to investigate the expression patterns of *EoSPLs* under cold stress [61]. Heatmap analysis was conducted using TBtools-II [50]. Analysis of the protein–protein interaction (PPI) network was performed utilizing the STRING database (https://cn.string-db.org/, accessed on 2 October 2024) [62].

### 4.9. Statistical Analysis

All statistical analyses and graphing were performed using IBM SPSS Statistics 27 and Origin 2024b. The least significant difference (LSD) test was used to compare the data.

## 5. Conclusions

In this study, nineteen *EoSPL* genes were identified and mapped within the centipedegrass genome, all of which were found to possess a complete SBP domain. Phylogenetic analyses revealed that genes within the same clade exhibit comparable gene structures and conserved motifs. Segmental duplication has significantly influenced the expansion of the *EoSPL* gene family in centipedegrass. Investigation of *cis*-acting elements suggests that *EoSPLs* are associated with plant development and responsive to phytohormones, as well as a multitude of stress conditions. Moreover, 10 *EoSPL* genes exhibited a targeted relationship with miR156. The *EoSPLs* are predominantly expressed in leaves and demonstrated diverse responses to abiotic stress. These findings establish a foundation for exploring the involvement of *SPL* genes in plant stress tolerance and provide a robust foundation for future investigations into the regulatory mechanisms of miR156/SPL modules, which can be instrumental in devising strategies to enhance the stress resistance of turfgrass species.

## Figures and Tables

**Table 1:** The information of 19 *EoSPL* genes in centipedegrass.

Gene Name	Gene ID	Molecular Weight (Da)	Theoretical pI	Instability Index	Aliphatic Index	Grand Average of Hydropathicity	Predicted Subcellular Location
*EoSPL1*	*evm.model.ctg22.106*	98,538.80	6.34	56.80	80.23	−0.353	Nucleus
*EoSPL2*	*evm.model.ctg62.167*	42,275.05	9.15	58.31	55.45	−0.482	Nucleus
*EoSPL3*	*evm.model.ctg112.24*	34,188.93	8.81	56.39	60.09	−0.369	Nucleus
*EoSPL4*	*evm.model.ctg201.47*	37,945.37	8.99	52.06	56.15	−0.363	Nucleus
*EoSPL5*	*evm.model.ctg199.108.1*	47,556.05	6.53	72.11	58.04	−0.360	Nucleus
*EoSPL6*	*evm.model.ctg191.89*	25,928.75	10.36	56.52	57.19	−0.456	Nucleus
*EoSPL7*	*evm.model.ctg191.88*	21,940.04	9.96	67.84	54.50	−0.530	Nucleus
*EoSPL8*	*evm.model.ctg385.98*	105,966.10	5.52	51.87	77.88	−0.341	Plasma Membrane
*EoSPL9*	*evm.model.ctg398.151*	46,814.39	9.23	67.20	57.84	−0.623	Nucleus
*EoSPL10*	*evm.model.ctg389.89*	47,434.04	9.41	37.67	65.23	−0.490	Nucleus
*EoSPL11*	*evm.model.ctg553.95*	94,630.41	5.49	49.03	84.22	−0.272	Nucleus
*EoSPL12*	*evm.model.ctg581.24*	46,244.15	7.58	64.74	54.12	−0.399	Nucleus
*EoSPL13*	*evm.model.ctg578.33*	122,131.35	6.82	55.04	76.24	−0.453	Chloroplast
*EoSPL14*	*evm.model.ctg577.71*	42,718.06	8.63	66.37	50.49	−0.638	Nucleus
*EoSPL15*	*evm.model.ctg704.64*	51,965.88	9.29	46.24	59.28	−0.544	Nucleus
*EoSPL16*	*evm.model.ctg701.30*	36,765.00	9.23	54.94	58.72	−0.718	Nucleus
*EoSPL17*	*evm.model.ctg700.26*	44,340.89	8.58	63.27	65.21	−0.301	Nucleus
*EoSPL18*	*evm.model.ctg780.110*	42,259.03	9.54	57.02	60.36	−0.429	Nucleus
*EoSPL19*	*evm.model.ctg784.113*	44,176.91	7.80	51.86	58.07	−0.592	Nucleus

**Table 2:** Ka/Ks of duplicated *EoSPL* gene pairs.

Duplicated Pair	Ka	Ks	Ka/Ks	Gene Duplication	Time (MYA)
*EoSPL2-EoSPL12*	0.4437	0.9810	0.4523	Segmental	75.4615
*EoSPL4-EoSPL14*	0.2990	0.8171	0.3659	Segmental	62.8538
*EoSPL5-EoSPL12*	0.2112	0.6782	0.3114	Segmental	52.1692
*EoSPL9-EoSPL17*	0.3501	0.5700	0.6142	Segmental	43.8462
*EoSPL10-EoSPL15*	0.2912	1.0690	0.2724	Segmental	82.2308
*EoSPL6-EoSPL7*	0.2652	0.3035	0.8739	Tandem	23.3462

## Data Availability

The original contributions presented in this study are included in the article/Supplementary Material. Further inquiries can be directed to the corresponding authors.

## Supplementary Materials

The following supporting information can be downloaded at https://www.mdpi.com/article/10.3390/plants14010062/s1: Table S1: the Ct value of qRT-PCR analysis; Table S2: Primers of the *EoSPLs* in qRT-PCR.
